# Integrating paleoparasitological, paleogenetic, and archaeological data to understand the paleoecological scenario of pre-Columbian archaeological site *Gruta do Gentio* II, Brazil

**DOI:** 10.3389/fmicb.2024.1505059

**Published:** 2025-01-20

**Authors:** Ludmila Gurjão, Lorrayne Brito, Ondemar Dias, Jandira Neto, Alena Mayo Iñiguez

**Affiliations:** ^1^Laboratório de Parasitologia Integrativa e Paleoparasitologia, Instituto Oswaldo Cruz, Fundação Oswaldo Cruz – IOC/FIOCRUZ, Rio de Janeiro, Brazil; ^2^Instituto de Arqueologia Brasileira (IAB), Rio de Janeiro, Brazil

**Keywords:** ancient DNA, coprolites, DNA barcoding, paleogenetic, paleoparasitology

## Abstract

Paleoparasitology and paleogenetics is the study parasites in ancient remains from latrines, mummified individuals, and coprolites, that is fossilized or desiccated feces. Paleoparasitological studies in Brazil began with analyses of coprolites from the *Gruta do Gentio* II (GGII) archaeological site, the oldest site related to the Una ceramist tradition (12,000 to 410 BP), Brazil. The GGII archaeological site contained numerous human burials, lithics, and cultural artifacts such as basketry, ceremonial ornaments, and unique pottery of the Una tradition. Coprolites of GGII were submitted to paleoparasitological, and paleogenetic analyses for parasite identification and coprolite origin. In addition, the archaeological characterization of the GGII site was integrated into paleo analyses for proposing a paleoecological scenario. Five taxa of parasites, including Ancylostomidae, *Echinostoma* sp., *Spirometra* sp., and *Trichostrongylus* sp., and three different morphotypes of Capillariidae were recognized in multiple coprolites that were distributed heterogeneously in several stratigraphical layers. The origin of coprolites was genetically defined as five species of mammals, humans, felines as *Panthera onca* and *Leopardus pardalis,* and marsupials as *Didelphis albiventris* and *Philander opossum*. This is the first study in Brazil that identified both, parasites and species of animals in Pleistocene/Holocene producers of coprolites with geographical and temporal information. The integration of paleoparasitology, paleogenetics, and archaeology is essential to propose paleoecological scenarios from the past of Brazil.

## 1 Introduction

Paleoparasitology is a discipline that concentrates on the detection and tracing of parasitic infections in ancient contexts (Ferreira et al., 2011). Its primary objective is identifying parasites within preserved remnants, such as sediments from the sacral region of buried individuals, latrines, and coprolites, which are fossilized or desiccated feces (Araújo et al., 2015). Parasitology studies symbiosis, broadly defined as the close association between two organisms, often of distinct species, while some definitions restrict symbiosis to mutually beneficial relationships, the term can refer to any close ecological interaction (Roberts et al., 2008). Dr. Luiz Fernando Ferreira and Dr. Adauto Araújo were pioneers in paleoparasitology, contributing significantly to the findings of parasites in archaeological materials from the Northeast to the Southeast regions of Brazil, using coprolite rehydration, spontaneous sedimentation, and light microscopy techniques. In 1980, Araújo and Confalonieri documented the presence of parasite eggs in animal remains dating back to 9,000 BP (Before Present) at the archaeological site of *Santana do Riacho* in southeastern Brazil. The study involved collecting fecal samples from small animals, primarily reptiles of the genus *Tropidurus*, inhabiting the vicinity of the archaeological site. These samples were analyzed to compare parasitological findings from both ancient and modern specimens. The analysis revealed the presence of helminth eggs identified as *Parapharyngodon sceleratus*, suggesting that the same species of helminth has been infecting reptiles in the region since pre-Columbian times (Araujo et al., 1980). In 1989, Ferreira et al. documented the presence of *Trichuris trichiura* eggs in human coprolites recovered from the *Furna do Estrago* archaeological site in northeastern Brazil. Dated to approximately 2,000 BP, human coprolites were found in association with burials and handcrafted artifacts. The study underscores the presence of trichurid infections among human pre-Columbian populations, expanding the paleodistribution of *T. trichiura* beyond the Brazilian southeastern region (Ferreira et al., 1989b).

Zoonotic parasites were identified in the intestinal contents of camelids in Peru belonging to Trichuridae, Fasciolidae, and Eimeriidae (Bailly et al., 2019). There is a diversity of three to seven taxa of parasites identified in the paleoparasitological analysis, indicating that the population was infected concomitantly with more than one parasite. The study provides information on the health and diet of Peruvian ancient camelids. Additionally, it discusses about the implication of the baseline health status of camelids in the region (Bailly et al., 2019). de Souza et al. (2018) identified a variety of parasites including *Trichostrongylus* sp., *Trichuris* sp., *E. vermicularis*, and *Eimeria macusaniensis* in human coprolites samples of the archaeological site *Lluta 57*, located in the *Lluta* Valley, Chile. The authors suggested that the three chronological periods, the Late Intermediate Period, the Late Period, and the Hispanic Contact Period, were associated with the diversity and frequency of parasites that infected the individuals inhabiting northern Chile, providing an opportunity to discuss the epidemiological transitions that took place (de Souza et al., 2018). Sianto et al. (2005) pointed out the presence of *Echinostoma* sp. in a human coprolite dating 600–1,200 BP, and proposed accidental infection caused by the consumption of tadpoles, planarians, and fishes that are *Echinostoma* spp. intermediate hosts.

Ancient DNA (aDNA) contributes to paleogenetics with the genetic information of parasites and hosts, diet, and environmental surroundings (Iñiguez, 2020). To obtain high-resolution results, paleogenetics became essential to study parasitic infections in archaeological individuals (Iñiguez, 2020). In addition to paleoparasitological analyses, paleogenetic studies were introduced in Brazil by Iñiguez and contributions (Iñiguez et al., 2003a,b, 2006). The authors demonstrated for the first time the direct aDNA detection of parasites without previous visualization of parasitic structures by paleoparasitological analysis, with the genetic identification of *E. vermicularis* from coprolites of different archaeologic sites from South America. In the study, two different haplotypes of pinworms were identified, a worldwide and a different lineage-specific to *Tulan* region, San Pedro de Atacama, Chile. Since *E. vermicularis* is a host-specific to humans, with a direct contact transmission, the identification of the 3,000-year-old *E. vermicularis Tulan* haplotype related to a different population in the region that was an important pre-Columbian trade route from the Pacific coast to the Andes Mountains (Iñiguez et al., 2006). By analyzing the intestinal microbiomes preserved in coprolites of ancient birds, researchers were able to identify the diverse array of microorganisms that once thrived within these birds, shedding light on their diets, health, and broader ecosystem dynamics (Boast et al., 2018). Boast et al. highlight the importance of intestinal microbiomes in understanding past ecologies and the impacts of species extinction.

Coprolites have broad acceptance as a source for relevant data on ancient environments and human activities, utilizing diverse methods and techniques (Blong et al., 2023). Researching the diet (Reynoso-García et al., 2023), coevolution of parasite and host (Halaçlar et al., 2023), and rebuilding of paleoenvironment and human-environment interactions, coprolites provide data on health and environmental dynamics, generally inaccessible via other analytical methodologies in multi-proxy research (Blong et al., 2023). Regarding paleogenetics, the analysis of aDNA can provide valuable information about the diets, microbiomes, and ecosystems of extinct organisms. Paleogenetics enables the reconstruction of ecological interactions and evolutionary adaptations, offering deep insights into the genetics and environments of ancient species (Blong and Shillito, 2021; Blong et al., 2023). Concerning aDNA from coprolites, the analysis reveals historical dietary compositions and microbiomes and contributes to a broader understanding of the evolutionary and ecological processes that have shaped biodiversity over time (Blong et al., 2023).

More recently, in molecular paleoparasitology in Brazil, researchers identified parasites such as *Ascaris* sp. infecting individuals from a coastal *sambaqui,* shellmound, archaeological site, dated up to 1,826 BP. By integrating paleoparasitological, paleogenetic, and microremains investigations, Iñiguez et al. (2022) revealed helminth infections in archaeological contexts. This research not only reveals information about the health of ancient inhabitants in the region but also provides insights into human mobility and social interactions during the pre-Columbian period along the Brazilian coast. Guedes et al. (2020) demonstrated *Ascaris* sp. aDNA sequences in an individual of African origin from the Brazilian colonial period, collected at *Pretos Novos* cemetery archaeological site in Rio de Janeiro. The *Pretos Novos* cemetery (1769–1830) served as a burial ground for enslaved Africans who died before being sold in the slave market or upon arrival in the city. The study revealed the possibility of researching historical human infections in the African population out of Africa. Guedes et al. (2018) conducted paleogenetic analyses in skull remains of young adults with no visible paleopathological signs of treponematoses. Ancient DNA sequences were recovered from two young adults, exhibiting the polymorphism characteristic of *T. p.* subsp. *pallidum* in a female, suggesting a case of syphilis infection. These findings underscore the importance of considering the epidemiological context and disease physiopathology in detecting infections in paleogenetic studies (Guedes et al., 2018).

**Figure 1:**
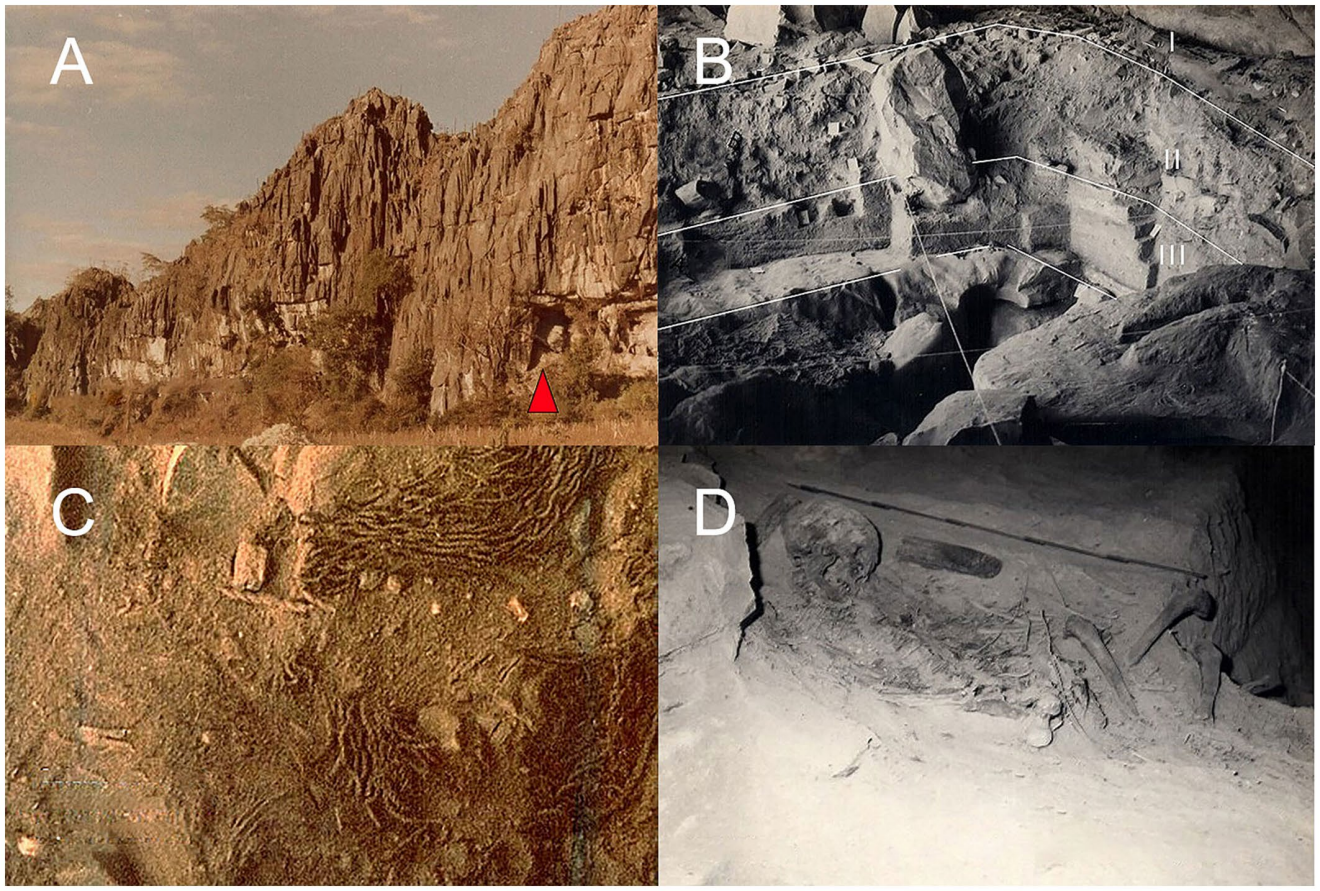
Images of the external and internal locations of the *Gruta do Gentio* II. (A) Limestone cliff with the cave entrance marked by a red triangle. (B) Excavation area of GGII, showing the stratigraphy: I, original ground level; II, Horticultural Horizon; and III, Archaic Hunter-Gatherer Horizon. (C) Evidence of bead necklaces and cordage in the Horticultural Horizon. (D) Primary human burial.

The archaeological site *Gruta do Gentio* II (GGII) is considered one of the oldest sites belonging to the Indigenous Una ceramist tradition of Brazil, with its origin in the Amazonian Forest. GGII was a shelter for different human groups that occupied the site alternating diachronically hunter-gatherers and ceramist horticulturalists (Sene, 2007) (Figures 1A–D). The presence of bonfires, basketry, ceramic, and lithic forms are cultural indications of these groups. The presence of numerous human burials in GGII was interpreted by the archeologists as a characteristic of a ceremonial archaeological site, a location that was utilized seasonally for burial rituals, which could extend over several weeks (Sene, 2007) (Figures 1C,D). Archaeologists uncovered primary and secondary burials during excavation, characterized by a notable abundance of buried individuals, occasionally with the co-occurrence of two or even three individuals within the same mortuary context (Cheuiche, 1992). The GGII archaeological site was discovered during an expedition organized by the archaeological research program of *Instituto de Arqueologia Brasileira* (IAB) in the São Francisco Valley – PROPEVALE, coordinated by Ondemar Dias Junior in 1973 (Dias et al., 1979). Material culture was found during four systematic phases of the excavations of the GGII site that occurred in 1970 and 1980. The elements of the GGII excavations allowed the proposal of two subsistence horizons: The hunter-gatherer of the *Paracatu* archaeological phase, dated between 12,000 and 8,125 BP, and the horticulturist dated between 410 and 3,490 BP, related to the Una ceramist tradition (Dias et al., 1979) (Figure 1B). The local microclimate favored the excellent preservation of materials including natural mummified individuals (Iñiguez, 2020).

According to Omran (2005), epidemiological transitions are based on essential theoretical analyses that highlight the following aspects: demographic, biological, sociological, economic, and psychological factors in the processes of changing patterns of health and disease in a population over time. This perspective seeks to understand how variables such as population growth, urbanization, economic development, and cultural transformations influence the epidemiological phenomena of a society (Omran, 1971, 2005). The first epidemiological transition occurred with an increase in infectious diseases, coinciding with the Neolithic Revolution, when humans shifted from a nomadic lifestyle, based on hunting and gathering, to a sedentary way of life centered around agriculture and animal domestication (Barrett et al., 1998; Omran, 2005). The first paleoepidemiological transition in the Americas is archaeologically unclear. Prehistoric Native Americans consumed a wide range of terrestrial and aquatic vertebrates and invertebrates, each carrying its own set of parasites capable of infecting humans. However, zoonotic parasites show little to no noticeable changes in diet-related parasitism during this period (Reinhard et al., 2013). The evidence suggests the presence of two distinct cultural horizons in GGII, each characterized by different subsistence strategies. Particularly when lithic artifacts found in the deepest and oldest strata give way to more complex cultural materials, these include exquisitely crafted basketry, ceremonial adornments, and indicators of early plant domestication. The emergence of ceramic production associated with the Una culture of Brazil exemplifies this transformation, highlighting the development of agriculture and more permanent settlements. This transition not only illustrates a shift in the economic base of the population but also signals profound changes in social organization. The two subsistence horizons in GGII are well-defined, providing a valuable opportunity to evaluate whether and how the epidemiological transition occurred.

Alongside the archeological research, GGII site was the subject of the first paleoparasitological study in Brazil. Ferreira et al. (1980, 1983) demonstrated *Trichuris trichiura*, and Ancylostomidae eggs in coprolites that were retrieved from the naturally mummified remains of an 8- to 9-year-old child dating from 3,490 ± 120 to 430 ± 70 BP (Smithsonian Radiation Biology Laboratory-SRBL). The GGII mummified individual studied, named Acauã, had a paleoparasitological analysis revisited that confirmed hookworm infection, and their human mitochondrial DNA (mtDNA) ancestry determined as belonging to subhaplogroup A2, one of the Amerindian autochthonous mtDNA lineage (Iñiguez, 2020). The findings of hookworm and whipworm infections suggest that the parasites were circulating in this ancient population of Brazil before European colonization. These geohelminth infections indicated that the increased population density of GGII facilitated the transmission of the parasite among its human definitive hosts (Ferreira et al., 1980, 1983). Ferreira et al. (1980) collected coprolites classified as human origin based on analyses of size, shape, colors, and odor after rehydration. Seven coprolite samples containing eggs of Ancylostomidae and *T. trichiura*, with the same measurements of *T*. *trichiura* eggs observed in ancient material of Szidat (1944), Pizzi and Schenone (1954), and Taylor (1955), attesting to the presence of the parasite species (Szidat, 1944; Pizzi and Schenone, 1954; Taylor, 1955).

The present study aims to integrate paleoparasitological evidence and the paleogenetic origin of coprolites with archaeological data, providing temporal data on human and animal parasitic infections in pre-Columbian Brazil. The study represents the first in the country to identify parasites and hosts/producers in coprolites from the Pleistocene/Holocene. The interdisciplinary approach underscores the importance of constructing paleoecological narratives.

## 2 Materials and methods

### 2.1 *Gruta do Gentio* II archaeological site

The archaeological site GGII (12,000 to 410 BP) is a sheltered area defined as a subterranean cave that exhibited topographical variations (Figures 1A,B), located at Var*gem Bonita* farm (16° 17′ 38″ S 46° 44′ 35″ W), *Santo Antonio do Boqueirão* locality, Unaí municipality, Minas Gerais state, southeastern Brazil (Dias et al., 1984). The archaeological site soil displays some punctual variations resulting from the process of formation and sedimentation of limestone, such as frequent rock collapses and the presence of sinkholes, causing unevenness in some stratigraphical layers (Sene, 2007) (Figure 1B). The excavation method employed was that of artificial level stripping, conducted every ten centimeters, by dividing the cave into square sectors of two meters each (Figure 1B). Numbered lines parallel to the cave entrance and alphabetized perpendicular lines were used (Sene, 2007). GGII site is classified as a ceremonial locus, with human material evidence, including food such as maize and peanuts, and customized ornaments like necklaces made of animal teeth, and handmade hammocks (Sene, 2007).

GGII archaeological site was characterized by two subsistence horizons, a hunter-gatherer dated between 8,125 BP and 12,000 BP defined by lithic artifacts, and a horticulturist horizon with dating of 3,490 ± 410 BP, where ceramics of Una tradition were recognized. The site was Carbon-14 dating (SRBL) and has the total temporal time sectioned in 3 periods, two horizons of subsistence associated with human occupation, and in between, an occupational gap of 3,500 BP. The hunter-gatherer horizon had material traces such as adornments made from bivalve shells, and the presence of animal and human remains. In the horticultural horizon, ceramic artifacts, food residues (tubers, maize, and other grains), basketry, a few bone fragments of animals, and numerous human burials were identified (Sene, 2007).

### 2.2 Samples

**Figure 2:**
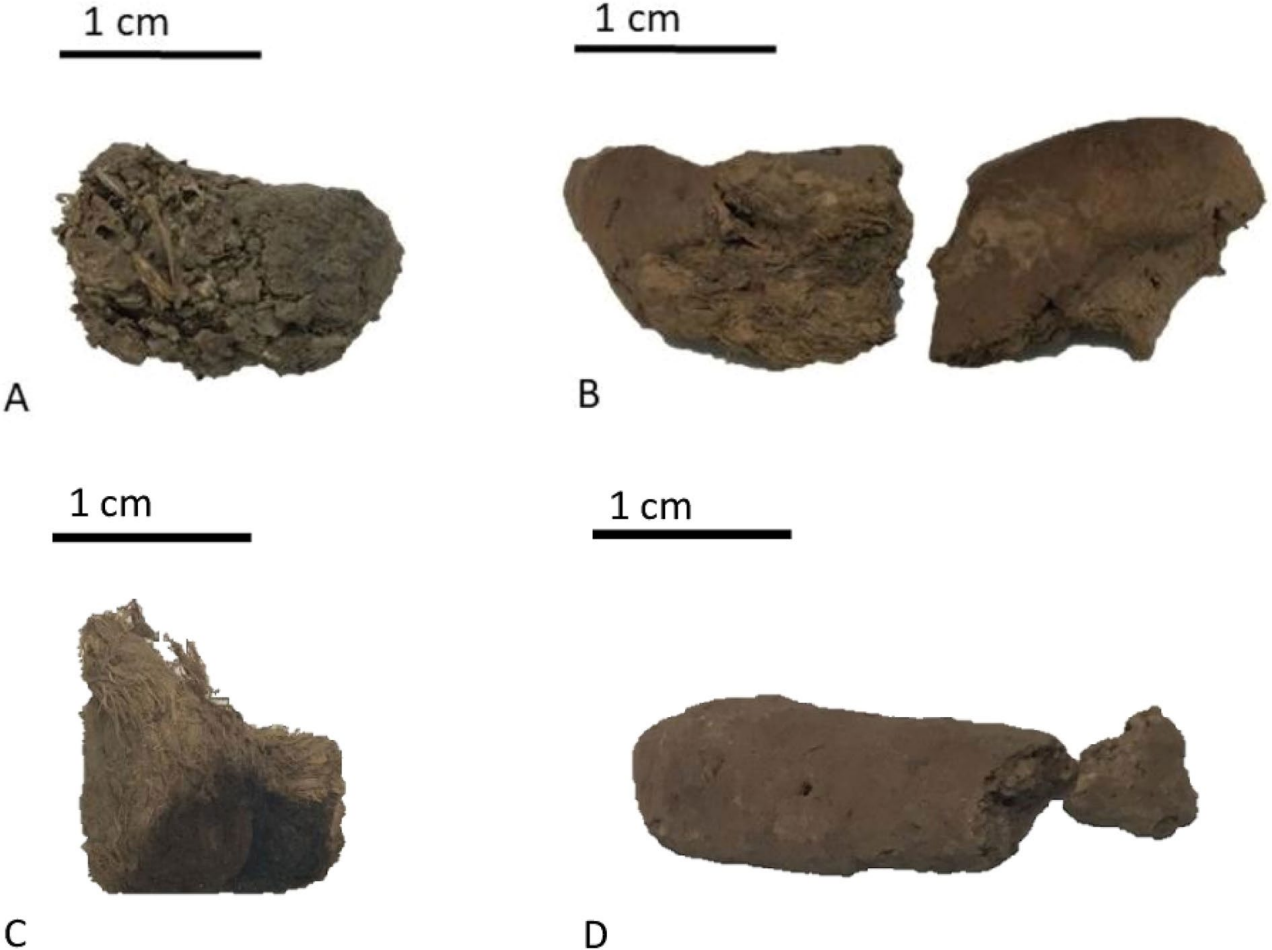
Coprolite samples of GG II classification following the classification of mammal feces of Chame (2003). (A–D) Coprolites identified with complex morphometry.

Coprolites analyzed in the present study were found dispersed across 1 to 9 stratigraphic layers spanning 16 archaeological sectors in layers up to 155 cm deep (Gurjão et al., 2024, in press). Samples were collected *in loco* from the internal, intermediate, and external cave areas between 1970 and 1980, by IAB archaeologists. The samples were curated and stored in the IAB collection, and some were collected for the first paleoparasitological studies. The conditions of preservation at the IAB facility were kept with plastic bags and plastic organizing boxes protected from light until Dr. Adauto Araújo and Dr. Luiz Fernando Ferreira collected some samples to conduct paleoparasitological analysis (Ferreira et al., 1980, 1983). In 2016, the Paleogenetic Laboratory (PL) team from the Laboratory of Integrative Parasitology and Paleoparasitology (LPIP) from the Instituto Oswaldo Cruz, Fundação Oswaldo Cruz (IOC/FIOCRUZ) under the responsibility of Dr. Alena Iñiguez conducted the paleogenetic collecting procedures at IAB facilities (Iñiguez, 2020). Samples were stored in the Paleoteca collection of the PL Laboratory LPIP/IOC/FIOCRUZ under the responsibility of Dr. Iñiguez in collaboration with the IAB Institute. GGII coprolites selected (n = 19) for the paleoparasitological and paleogenetic analysis presented variable morphologies and morphometries that indicate human, or mammal origin (Figure 2) according to the study performed by Gurjão et al. (2024). GGII coprolites were also analyzed following the methodology proposed by Chame (2003), which classifies feces into nine distinct morphological groups (Table 1). Carnivores typically produce cylindrical, segmented feces, often tapered at one or both ends, with a relatively firm consistency. In this group, the reference mentions the possibility of felids and canids as representatives (Group I). Herbivores produce generally rounded or pellet-shaped feces, with a softer texture due to the higher fiber content of their diet (Groups, I, II, III, IV, and V). Group VI, described by flattened feces that accumulate in circular piles, is associated with species from the Bovinae family. Group VII is constituted of single riniform feces, related to Equinidae. Group VIII is represented by big and cylindrical feces that characterize large ungulates, and group IX, with amorphous feces varying from cylindrical to rounded, with no consistent or distinctive features that can be linked to any specific group. In this group feces from primates, marsupials, and insectivores are included according to Chame (2003).

**Table 1:** Paleoparasitological results of GGII coprolites.

Dating	Coprolite ID	Layer (cm)	Sector	CoproM	Group	Possible origin	PaleoG	PaleoP	Length (μm)	Width (μm)	Plug length	Plug width
3,490 ± 410 BP	GG01	10/20	ND3	CX	I	Mammal	*P. onca*	*C. venusta*	51.672	25.032	6.444	2.348
3,490 ± 410 BP	GG15	0/10	ND3	CX	IX	Mammal	*D. albiventris*	*A. myoxinitelae*	76.923	30.312	9.739	7.192
3,490 ± 410 BP	GG15	0/10	ND3	CX	IX	Mammal	*D. albiventris*	*A. myoxinitelae*	60.740	41.027	NI	NI
3,490 ± 410 BP	GG15	0/10	ND3	CX	IX	Mammal	*D. albiventris*	*A. myoxinitelae*	63.556	43.221	NI	NI
3,490 ± 410 BP	GG15	0/10	ND3	CX	IX	Mammal	*D. albiventris*	*A. pulchra*	59.952	39.741	NI	NI
3,490 ± 410 BP	GG15	0/10	ND3	CX	IX	Mammal	*D. albiventris*	*A. pulchra*	41.204	52.644	NI	NI
3,490 ± 410 BP	GG15	0/10	ND3	CX	IX	Mammal	*D. albiventris*	*A. pulchra*	58.636	41.086	NI	NI
8,125 to 7,295 BP	GG30	30/40	NB4	CX	IX	Mammal	*H. sapiens*	*Trichostrongylus* sp.	84.46	47.200	NA	NA
8,125 to 7,295 BP	GG32	20/30	NB4	CX	IX	Mammal	*H. sapiens*	*Trichostrongylus* sp.	80.553	42.828	NA	NA
3,490 ± 410 BP	GG38	10/20	OA0	CX	IX	Mammal	*L. pardalis*	*Echinostoma* sp.	123.389	53.356	NA	NA
3,490 ± 410 BP	GG38	10/20	OA0	CX	IX	Mammal	*L. pardalis*	*Echinostoma* sp.	98.929	63.424	NA	NA
3,490 ± 410 BP	GG38	10/20	OA0	CX	IX	Mammal	*L. pardalis*	*Spirometra* sp.	60.27	43.284	NA	NA
3,490 ± 410 BP	GG38	10/20	OA0	CX	IX	Mammal	*L. pardalis*	*Spirometra* sp.	61.881	43.519	NA	NA
3,490 ± 410 BP	GG38	10/20	OA0	CX	IX	Mammal	*L. pardalis*	*Spirometra* sp.	64.029	36.64	NA	NA
3,490 ± 410 BP	GG38	10/20	OA0	CX	IX	Mammal	*L. pardalis*	*Spirometra* sp.	60.190	37.543	NA	NA
3,490 ± 410 BP	GG38	10/20	OA0	CX	IX	Mammal	*L. pardalis*	*Spirometra* sp.	64.991	34.021	NA	NA
3,490 ± 410 BP	GG74	50/60	LB0	FL	IX	UN	*H. sapiens*	Ancylostomatidae	67.453	34.913	NA	NA

Information about dating, layer, sector, coprolite classification, paleogenetic and paleoparasitological results, and morphometry of helminth eggs. CoproM: Morphology of coprolites as defined by Gurjão et al. (2024). CX, Complex; FL, Flat. Group: The morphology coprolite group was defined following (Chame, 2003). Group I—Carnivorous morphology coprolite. Group IX—Mixed fecal shapes coprolites. Possible Origin: possible origin of coprolites as defined in Gurjão et al. (2024). UN, Undetermined; PaleoG, Paleogenetic results of coprolite productor/host origin; PaleoP, Paleoparasitological results; C., Capillaria. A., Aoncotheca; NI, Identified; NA, Not applicable. Only positive results from the paleoparasitological and paleogenetic analyses are shown.

### 2.3 Paleogenetic measures

During paleogenetic collection, the sample requires the use of sterile and disposable materials, with the Personal Protective Equipment (PPE) worn by the handler to be changed for each coprolite sample. The archaeological samples were preserved immediately after collection protected from light and kept on ice in sterile containers. They were transported to the Paleogenetics laboratory at 4°C and held at −20°C until paleoparasitological and paleogenetic analyses were performed (Iñiguez, 2020).

The PL facility maintains strict entrance control and has a pre-room for the dressing of all necessary PPE: coat, mask, shoe covers, and cap. Its interior is subdivided into small specific rooms for each specific method, paleoparasitological analysis, material preparation for aDNA extraction, and PCR procedures. Surface decontamination of metallic surfaces is done using 70% alcohol, and non-metallic surfaces are decontaminated using 1% sodium hypochlorite, followed by irradiation with UV light for 15 min in the designated hood or cabinet to be used (Iñiguez, 2014, 2020). The operators weekly clean the laboratory themselves. As an authenticity measure and prevention against modern DNA contamination, the authenticity criteria consist of the absence of detectable PCR products (pPCR) in PCR-negative controls. PCR-positive controls are never used in the Paleogenetic Laboratory. The PL manages a database of mtDNA haplotype ancestry for each operator or archaeologist involved in the scientific project in the course to control and monitor access to the studied material and laboratory. All external surfaces of the coprolites were removed with a scalpel and exposed to UV light for 15 min on all surfaces. Subsequently, only the core of the coprolite was utilized in this current study (Iñiguez, 2020).

### 2.4 Paleoparasitological analyses

Paleoparasitological analyses were conducted to identify the presence of parasite vestiges by light microscopy. About 5 grams of coprolites (Supplementary Table S1), randomly subsampled, were submitted to rehydration following Callen and Cameron (1960) technique, with modifications of a 48 h period rehydration, instead 72 h and at 5° to 10°C, instead room temperature, to avoid microorganisms proliferation. After that, for parasite concentration, procedures of spontaneous sedimentation technique by Lutz (1919) were applied, following our standardized protocol with modification to 3 h of sedimentation.

About 20 slides equal to 200 μL of coprolite sediment were submitted to paleoparasitological analysis by light microscopy. For parasitological identification morphological and morphometrical characterization of parasite eggs was conducted using PHYSIS microscope and 10×, 40×, or 100× lens, according to the classical parasitology literature of gastrointestinal parasites (Taylor et al., 2017; Rey, 2008; Cimerman and Franco, 2019; Neves and Neto, 2019). The approach proposed by Borba et al. (2021a) was applied specifically for capillariids, consisting of an updated taxonomy classification based on the egg structure and a reference dataset that included morphological and morphometrical characteristics of parasite eggs and ecological information of species of capillariids deposited in helminthological institutional collections from the New and Old World. The authors chose Artificial Intelligence/ Machine Learning (AI/ML) algorithms from the WEKA 3.8 program, which provides decision trees similar to the taxonomic keys used by systematics specialists to distinguish biological species (Witten and Frank, 2005). The IA/ML classification algorithms generated models that were evaluated using cross-validation (Borba et al., 2021a).

### 2.5 Paleogenetic

Coprolite sediment was submitted to DNA extraction using the QIAamp DNA Investigator kit (Qiagen) with modification of physical digestion by liquid nitrogen and double chemical digestion by proteinase K 20 mg/mL (Invitrogen) (Iñiguez, 2020). REPLI-g Single Cell Kit (QIAGEN) was used as a pre-PCR step to improve the amplification of DNA. The DNA was quantified using the Quantus™ Fluorimeter (Promega). PCR reactions using a DNA Barcoding approach targeting the 12S ribosomal DNA (12S rDNA) region were conducted according to Kitano et al. (2007) to identify coprolite origin/producer. The 12S rDNA genetic target is a DNA Barcoding marker that allows robust and reliable vertebrate species discrimination.

The PCR reaction with a final volume of 25 μL contained 2 U of *GoTaq* G2 Hot Start (Promega), 1X buffer, 3 mM MgCl_2_, 0.4 mM dNTPs, and 10 μM of each primer. The cycling consisted of an initial denaturation at 95°C for 3 min, followed by 45 cycles of 30 s at 95°C, 30 s at 55°C, and 30 s at 72°C, with a final extension for 10 s at 72°C. The reactions were performed on a SimpliAmp*™* Thermal Cycler (Applied Biosystems). Electrophoresis analyses were conducted in the Molecular Biology Laboratory of the Trypanosomatid Biology Laboratory at the Oswaldo Cruz Institute (LABTRIP/IOC), which is geographically distant from the PL/IOC Lab. Electrophoreses were performed using Agarose Low Melting 3% (Invitrogen), molecular ladder 50 pb (Ludwing), and GelRed Nucleic Acid Gel Stain (BIOTIUM). DNA purifications were conducted employing MinElute PCR Purification Kit (Qiagen) following the protocol of manufacturers. The sequencing reactions used the Kit Big Dye Terminator v. 3.1 (Applied Biosystems, Foster City, California) in the sequencing facility RPT01A/IOC-Fiocruz (Applied Biosystems ABI 3730 sequencer). The sequence analyses were performed using Lasergene Seqman v.7.0.0 (DNASTAR, Madison, Wisconsin) and Bio Edit v. 7.0.4 (Department of Microbiology, North Carolina State University, Raleigh, North Carolina). BLAST searches were executed at NCBI^1^ to identify the obtained aDNA sequences.

## 3 Results

### 3.1 Paleoparasitology

**Figure 3:**
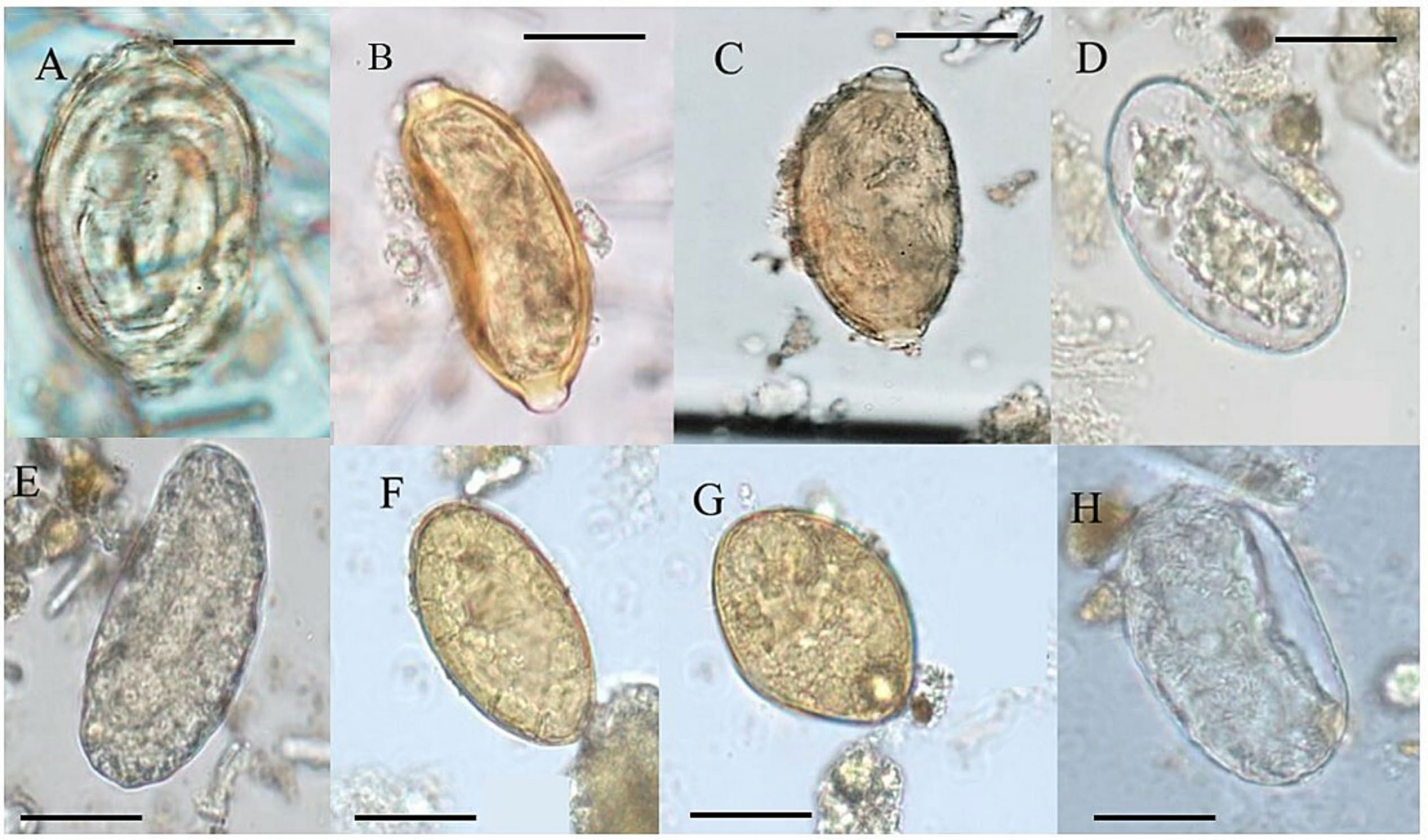
Helminth eggs identified in coprolites of GGII. (A) Morphotype 1 of Capillariidae; (B) Morphotype 2 of Capillariidae; (C) Morphotype 3 of Capillariidae; (D) Ancilostomatidae; (E,H) *Trichostrongylus* sp.; (F) *Echinostoma* sp.; (G) *Spirometra* sp. (100×).

Based on the criteria of the mammal fecal characteristic of Chame (2003), we classify the samples from the present study for morphology and morphometry related to I-IX groups (Table 1). Paleoparasitological analyses showed 12 positive coprolite samples of 19 with different morphotypes of helminth eggs (Table 1). Eggs of Ancylostomidae, *Spirometra* sp., *Echinostoma* sp., *Trichostrongylus* sp., and Capillariidae, with 3 different morphotypes were identified (Figure 3; Table 1).

Morphometric measurements for parasitological identification were taken according to the classic literature on parasite classification (Taylor et al., 2017; Cimerman and Franco, 2019; Neves and Neto, 2019; Rey, 2008). Ancylostomidae eggs were classified according to literature those described as measuring 60 μm in length for *Ancylostoma duodenale* and 70 μm for *Necator americanus* and 40 μm width, having an oval shape, thin and transparent shell, with a wide and clear space between the shell and the content of the eggs (Neves and Neto, 2019). Following the egg measurement in this study of 60–75 μm length by 35–40 μm width, *Trichostrongylus* sp. eggs are in agreement with the literature, with the very same morphology as Ancylostomatidae but differ in the size of egg length (Table 1). In this study, the egg measurements for Ancylostomatidae are 67 μm in length to 34 μm in width. *Echinostoma* sp. eggs were in agreement with the literature with between 80–135 μm length by 55–80 μm width (Taylor et al., 2017), with yellowish eggs, non-embryonated, and a thin shell. *Spirometra* sp. eggs were characterized by yellowish eggs, non-embryonated eggs, measuring 60 μm length and 40 μm width, according to classic literature (Taylor et al., 2017). Capillariidae eggs display a barrel-shaped morphology, ranging from circular to elongated and polar plugs (Spratt, 2006). Eggs measuring 54 to 64 μm in length and 29 to 33 μm in width were identified using the parasite egg chart according to Neves and Neto (2019) and Taylor et al. (2017). Additional capillariid egg measurements were considered including the base of the polar plug width, the base of the polar plug height, and shell thickness (Borba et al., 2021a). To determine capillariid species the classification of external ornamentations was considered as smooth, punctuated, reticulated type I, and reticulated type II (Borba et al., 2021b). To determine the most reliable set of information for species identification, the parameters MM (morphology and morphometry) + H (host) + GL (geographical location) were tested in combination for the construction of decision trees generated by AI/ML (Borba et al., 2021a).

The first morphotype identified in GGII01 was classified as *Capillaria venusta* or *Capillaria exigua*. The second morphotype, identified in GGII1501 was classified as *Aoncotheca myoxinitelae,* and samples GGII33 and 51, the fourth and fifth eggs were identified as *C. venusta* when considering MM + GL or *C. exigua* considering only MM. The third morphotype identified in GGII15-01 is compatible with *A. myoxinitelae* according to MM + H + GL, but when considering only MM, GGII15-01 is classified as *B. resecta.* GGII15-02 considering MM + H + GL determined the species *A. pulchra.* Certain Capillariidae eggs may undergo the loss of their external plugs as a result of taphonomic processes, exemplified by the occurrences observed in GGII15-01 sample. Consequently, a subset of Capillariidae eggs could not be morphometrically characterized with their respective plugs.

### 3.2 Paleogenetic

Through the DNA Barcoding approach, five aDNA species of animals were identified from coprolites as producers/hosts (Table 2). According to sequence references available on GenBank, jaguar, (*Panthera onca*), ocelot (*Leopardus pardalis*), gray four-eyed opossum (*Philander opossum*), white ear opossum (*Didelphis albiventris*), and human. DNA sequencing of jaguar had genetic identity up to 98.48 and 100% of query coverage according to GenBank, DNA sequencing of white-ear opossum had genetic identity up to 99.06 and 100% of query coverage, as well as *Homo sapiens* with 100% of and 100% query coverage and ocelot with a genetic identity of 99.54% and query coverage of 100% (Table 2).

**Table 2:** Paleogenetic results of positive samples identified in paleoparasitological analyses.

Coprolite ID	Paleoparasitological result	Paleogenetic result	Genetic identity	Query covering	GenBank reference sequence
GG01	*Capillaria venusta*	*Panthera Onca* (jaguar)	98.48%	100%	KX419751
GG15	*Aoncotheca myoxinitelae* and *Aoncotheca pulchra*	*Didelphis albiventris* (White-ear opossum)	99.06%	100%	FI28227
GG74	Ancylostomatidae	*Homo sapiens*	99.53%	100%	MG571158
GG30	*Trichostrongylus* sp.	*Homo sapiens*	100%	100%	MG571158
GG32	*Trichostrongylus* sp.	*Homo sapiens*	99.33%	100%	MG571158
GG38	*Spirometra* sp./ *Echinostoma* sp.	*Leopardus pardalis* (ocelot)	99.54%	100%	KP202284
GG58	Ancylostomatidae	*Homo sapiens*	99.32%	100%	MG571158
GG02	IT Larvae	*Homo sapiens*	99.48%	100%	MG571158
GG13	IT Larvae	*Homo sapiens*	100%	100%	MG571158
GG41	IT Larvae	*Philander opossum* (Gray four-eyed Opossum)	98.51%	100%	KJ868146
GG48	IT Larvae	*Homo sapiens*	99.33%	97%	MG571158
GG52	IT larvae	*Homo sapiens*	100%	100%	MG571158

IT Larvae, Indeterminate Taxonomy Larvae.

## 4 Discussion

Paleoparasitological analyses combined with morphological identification of coprolites, and paleogenetic analyses have become more commonly used in coprolites studies of the New World (Beltrame et al., 2016; Velázquez et al., 2020; Reynoso-García et al., 2023; Tietze et al., 2023). Studies identified the origin of coprolite samples focus primarily on the morphological and morphometrical features associated with environmental, or isotopic and biochemical data (Blong and Shillito, 2021). The present study focused on the paleoparasitological and paleogenetic analysis of GGII coprolites. We integrated paleoparasitological coprolite analyses to identify the presence of gastrointestinal parasites, with paleogenetic analyses to identify genetically the origin of coprolites. In paleoparasitological studies, the possible origin of coprolites is identified through morphological and morphometrical features along with diet composition analysis (Araújo et al., 2015). Chame (2003) published measures and figures of different morphotypes of feces associated with Brazilian mammals, and corroborates with scientific data about animal excreta, to compare with coprolites for primary analysis related to origin. Still, according to Chame (2003), shapes and measurements of coprolites can help to differentiate species within the same mammal group (Chame, 2003). Since the study of Chame (2003), groups that research parasites in animal or human coprolites used the guide to coprolites origin (McDonough, 2019; Fugassa et al., 2021; Ramirez et al., 2021; Agustín et al., 2021; Beltrame et al., 2017; Hunt et al., 2012). In the present study, following the fecal morphological groups described by Chame (2003), we observed the morphology of two proposed groups, Group I and Group IX. Group I corresponds to the fecal morphology primarily of carnivores, while Group IX refers to mixed morphology, with origins from various animal classes. Group I morphology was identified in sample GG01, which later yielded paleogenetic results confirming it as a coprolite from *Panthera onca*. Meanwhile, the remaining coprolites from felines, marsupials, and humans were classified under Group IX, which includes mixed shapes and sizes potentially from opossums, primates, armadillos, and insectivores. These results are consistent with and support the subsequent paleogenetic identification of coprolites from carnivores (*Panthera onca* and *Leopardus pardalis*), marsupials (white-eared opossum), and primates (humans).

In this way, morphological identification of coprolites is crucial for rapid screening and proposing potential origin and their parasites, as demonstrated by Borba et al. (2019). The authors demonstrated that when the host or the producer of the coprolite is known, the chances of identifying the parasite are higher (Borba et al., 2019). However, the ongoing taphonomic process could lead to multiple fragmentations that challenged the characterization of coprolites, resulting in low specific identification of producers. While registering coprolite characteristics and morphological data can provide valuable information for cataloging coprolites, our study relied on paleogenetic applications for coprolite identification. GGII coprolites were analyzed as an initial screen for producer identification, following the dataset of Chame (2003) and the datasheet proposed by Jouy-Avantin et al. (2003), considering coprolites morphology and morphometry (Gurjão et al., 2024). In many cases, irrespective of the taphonomic processes involved, morphological investigations of coprolites do not afford precise taxonomic identification of animals to the species level of the basic classification of living organisms (Hunt et al., 2012). Although DNA detection has become increasingly sensitive, there remains much to be understood about taphonomy, encompassing both natural and anthropogenic processes that directly influence the archaeological record, as well as transformative soil factors affecting the preservation of funerary contexts associated remains (Knüsel and Robb, 2016).

Paleogenetic studies of animal coprolites are focused on samples related to extinct animals (Hunt et al., 2012; Boast et al., 2018). In South America, the investigation into the origin of animal coprolites from the Pleistocene–Holocene through molecular analyses has intensified, as demonstrated by the study conducted by Petrigh and Fugassa (2017) that identified *Puma concolor* coprolite dated around 5,120 BP with *Calodium* sp. and *E. macusaniensis* oocysts. Fugassa et al. (2018) performed a PCR in coprolite samples from the Campo Cerda 1 archaeological site, which is dated to 1,700–2,900 BP, with primers specific to canids and showed a molecular identity of 97% with a sequence of gray fox. Genetic identification along with morphology and morphometry of animal coprolites in Brazil are not too often studied. The present study is the first identification of coprolite origin through paleogenetic analysis in an archaeological site from Brazil, belonging to 5 distinct species of animals (Table 2). The animals identified are found in the *Cerrado* Brazilian biome nowadays, the richest savanna in biodiversity in the world and the second-largest biome exclusive in the country (Costa et al., 1981). These animals are found not only currently, but there are records of carnivorous and marsupial bones from paleontological studies in the Pleistocene–Holocene periods, indicating that the faunal constitution did not change along the extension years (Costa et al., 1981; Galetti, 2019).

Paleoparasitological analyses demonstrated 5 taxa of parasite eggs in coprolite of humans, marsupials, and felids. Humans serve as definitive hosts for two of the identified parasite taxa, Trichostrongylidae and Ancylostomatidae (Neves and Neto, 2019). *Ancylostoma duodenale and Necator americanus* are monoxenous parasites that exclusively complete their life cycle and achieve infectivity within the human host (Stoll, 1946). There are only two Ancylostomatidae species that cause intestinal hookworm disease in humans and their eggs pass through the feces. Conversely, *Trichostrongylus* species parasitize mammals and exhibit a heteroxenous nature, capable of completing their evolutionary and infectious cycle in multiple hosts, including humans, who may be either definitive or intermediate hosts (Holmes, 1985). In humans and animals, infestation by *Trichostrongylus* spp. typically arises from ingesting contaminated water or food (O’Connor et al., 2006). The viability of the parasite is intricately linked to the ideal temperature and moisture conditions in which the free-living larval stages develop (Holmes, 1985). From goats and horses to avians and small mammals, nematodes belonging to the Trichostrongylidae, specifically the genus *Trichostrongylus*, impact the respiratory and digestive systems of a diverse array of animal species. Predominantly found among herbivores, including humans. In both humans and animals, trichostrongylosis is related to adult parasites in the small intestine, which can induce severe enteritis. These parasites dwell in the intestinal epithelium throughout their developmental stages. This leads to appetite loss, diarrhea, depletion of endogenous proteins, compromised food absorption, and abdominal discomfort (Souza et al., 2013; Gholami et al., 2015).

In paleoparasitological literature, *Trichostrongylus* sp., capillariids, ancylostomid, and *Spirometra* sp. eggs are reported in the New World, including Brazil (Ferreira et al., 1980, 1983, 1989a, 1989b; Araújo et al., 1984; Borba et al., 2021b). Until the worldwide paleoparasitological revision of Gonçalves et al. (2003), the findings of zoonotic parasites such as *Trichostrongylus* spp., were restricted to the New World and related to the consumption of wild animals such as cervids, different from ancylostomids parasites, related to human aggregation and population growth. GGII is a cave measuring 200 m^2^ and considering that human groups frequented the site collectively, as it was used for burial ceremonies lasting days to weeks, there is a high likelihood of overcrowding and ease of infection by parasites related to aggregation. Hookworms have a greater potential for transmission in locations where food and latrines are used in the same space (Hotez, 2008). de Souza et al. (2018) reported the presence of eggs from this parasite in coprolites dating back to the pre-Inca period at an archaeological site in northern Chile. Authors report that during the Late Intermediate Period or Pre-Inca Period, *Trichostrongylus* sp. was the only parasite detected, indicating a consistent source of human infection across time. Thus, the authors report that the expansion of the Inca civilization and the early lifestyle during the Hispanic Contact Period (HCP) might have impacted transmission dynamics, increasing human parasitic infections (de Souza et al., 2018). Among the species of *Trichostrongylus* known to infect humans, *T. colubriformis* and *T. orientalis* are the most commonly found worldwide (Wallace et al., 1956; Gholami et al., 2015). The success of parasite development is related to the optimal temperature and humidity conditions in which the free-living larval stages are found (Souza et al., 2013). In Brazil, species of *Trichostrongylus* were identified as parasitizing animals such as sheep (Almeida et al., 2010), bovids (Lima, 1998; Santos et al., 2010), goats (Radavelli et al., 2014), and even rodents (Santos et al., 2016).

It is possible to see primarily zoonotic parasites in the present study, such as *Spirometra* sp., and *Echinostoma* sp. in the horticulturist layers, and *Trichostrongylus* sp. in the hunter-gatherer layers. The study revealed the oldest finding of *Trichostrongylus* sp. eggs in Brazilian coprolites, dating the human infection to 7,925–8,125 BP. The helminth trematode *Echinostoma* sp. was found in paleoparasitological studies of Brazil in coprolite recovered from the pelvic region of an individual at the archaeological site of *Lapa do Boquete* in southeastern Brazil (Sianto et al., 2005). The authors report that the discovery of *Echinostoma* sp. in a pre-Columbian individual demonstrates that this infection existed among ancient groups due to dietary habits that included the consumption of aquatic intermediate hosts involved in the parasitic evolutionary cycle (Huffman and Fried, 1990). Digenetic trematodes of the genus *Echinostoma* inhabit the intestines of mammals and birds, with occasional infections in humans, who excrete along with their feces into lakes or streams of birds or mammals (Fried, 2001). Once in the water, fertilized eggs take up to 3 weeks to develop into miracidia. These miracidia then invade snails, typically of the species *Biomphalaria*, where they transform into cercariae. Cercariae remain encysted in the kidneys until they are consumed by the second intermediate host, which can include amphibians, fish, and other snail species (Slifko et al., 2000; Fried, 2001). The parasite reaches its definitive host when mammals and birds ingest the second intermediate host containing metacercarial cysts. Inside the intestine, the cysts mature into adult parasites, perpetuating the life cycle. Most species do not show host specificity, *Echinostoma caproni* and *E. trivolvis* have been identified as parasitizing rodents, shrews, domestic ducks, and swans (Meyer, 1970). Although there are no current records of felids in Brazil infected with *Echinostoma* species, Sianto et al. (2016) identified an egg with measurements corresponding to *E. erraticum* and *E. neglectum* in felid coprolites dated by 1,040 ± 50 to 1,730 ± 70 BP. Samples were collected in the archeological site of *Furna do Estrago* Northeast Brazil. The authors suggested that could be characterized as a case of false parasitism, when the felid may have preyed upon a bird already harboring the adult stage of the parasite (Sianto et al., 2016).

The species *Spirometra mansonoides* is most prevalent in the American continent, primarily infecting felines and canids (Meyer, 1970). The life cycle involves two intermediate hosts. Initially, an aquatic invertebrate, such as a copepod crustacean, hosts the larval stage known as procercoid (Mueller, 1974). Subsequently, the second intermediate host, which can be an amphibian, reptile, fish, or small mammal, leads to the development of the procercoid stage into plerocercoid (Mueller, 1974). When it reaches its definitive host, adult parasites can trigger inflammatory processes through mechanical activity in the intestine. Infections by *Spirometra* spp. are contracted through ingestion of water contaminated (Meyer, 1970; Mueller, 1974). Humans become part evolutionary cycle as accidental hosts, as the plerocercoid larvae can cause a disease known as sparganosis (Wiwanitkit, 2005; Mentz et al., 2011). In Brazil, *Spirometra* spp. eggs were found in feces or adult parasites were discovered in the intestines of various animals, including small mammals like opossums and jaguars from the Atlantic Forest (Mueller, 1974; Antunes, 2005; Borges-Pereira et al., 2008).

Species of *Spirometra* and *Echinostoma* are parasites that have one obligatory phase of development in the water, the presence of these parasites in ocelot coprolite may characterize true or false parasitism. True parasitism occurs because felines are part of the definitive cycle of the parasite and can feed on intermediate hosts like fish and amphibians infected by species of *Spirometra* and *Echinostoma.* In the case of the ocelot, its diet composition showed that amphibians were the most abundant (50%), followed by mammals (31.25%), reptiles (12.50%), and birds (6.25%), suggesting that the ocelot from which the coprolite originated could have fed on any type of intermediate host infected with *Echinostoma* and *Spirometra* (Shuingues et al., 2019). There is also the possibility of false parasitism, in which the ocelot may have consumed prey that is itself a definitive host in the life cycle of *Spirometra* and *Echinostoma,* or prey that does not act as an intermediate host in the life cycle of the parasites. Small mammals such as rodents and marsupials serve as definitive hosts for species of *Echinostoma*, such as *E. echinatum, E. luisreyi*, and *E. paraensei* (Meyer, 1970).

The false parasitism is also proposed since members of Felidae usually do not feed on animals that serve as intermediate hosts for *Echinostoma* spp., such as anurans and snails. This prevents the parasite from completing its evolutionary cycle, thereby precluding it from being characterized as the definitive host (Meyer, 1970; Mueller et al., 1975; Huffman and Fried, 1990). Rodents, particularly species from the Cricetidae, constituted the majority of their prey. Birds were the second most frequent prey item, followed by reptiles, including lizards and snakes. This dietary flexibility underscores the generalist predator role of ocelots, capable of adapting their feeding habits to the availability of prey in their environment (Abreu et al., 2008). Bodies of water are found in the area close to the rocky outcrops of the GGII cave (16° 17′ 38″ S 46° 44′ 35″ W)^2^ in the form of small waterfalls, ponds, wetlands that are currently used by farms in the region. The water sources in the vicinity of GGII cave were indicated by the presence of archaeological artifacts as ornaments like bracelets and necklaces made from bivalve shells and also by the results of the present study that demonstrated the circulation of parasites that require an aquatic environment to develop into their infective stages.

Sianto et al. (2014) documented the first occurrence of *Spirometra* species in felines in pre-Columbian Brazilian times. The present work is the second to report *Spirometra* species eggs in the southeast region of pre-Columbian Brazil. The geographical distance and distinct climatic and vegetal features of each biome suggest that the parasite had widespread distribution in ancient times, likely across various host species. The potential for this parasite to circulate among prehistoric populations, both animal and human, cannot be overlooked. Regarding *Spirometra* species, felids play an important role in the developmental cycle as the definitive host (Meyer, 1970). Consequently, the identification of eggs from this parasite in the ocelot coprolite suggests true parasitism, given their potential to feed on fish harboring the infective stages.

According to Gurjão et al. (2024), the morphometries and morphologies of coprolites, including attributes such as color and volume, were primarily associated with mammals, both carnivores and herbivores, where complex coprolites indicating a mixed origin. It was observed that sample GG01, identified as a carnivore coprolite, corroborated the result of *Panthera onca*, and sample GG38, identified as a mammal, confirmed the result of *Leopardus pardalis* in the present study. Additionally, sample GG15, identified as a possible marsupial coprolite (Table 1), also corroborated the result of *Philander opossum* in the current study. The remaining coprolites analyzed presented a complex and flat morphology and morphometry, making it challenging to determine their origin based on preliminary analysis (Gurjão et al., 2024, in press). Samples GG01, 15, and 38 were identified in stratigraphic layers associated with the horticultural horizon, with dates up to 3,490 BP. The presence of coprolites from these animals in a more recent subsistence horizon suggests that animals such as *Panthera onca, Leopardus pardalis*, and *Didelphis albiventris* roamed inside the cave, potentially being associated with the ceremonial practices of the human population that frequented the interior of GGII cave.

The paleogenetic and paleoparasitological results of the present study, analyzed with the archaeological data, make it possible to propose a GGII paleoecological panorama. GGII is classified as a ritualistic archaeological site, where people utilized the space for several days until the ritual was complete (Sene, 2007). The presence of food remains or items used in the rituals, the storage, accumulation, and cooking of food products, could have facilitated the presence of animals, making possible an association with several mammals like rodents, peccary, armadillos, and canids, animals reported in the *Cerrado* biome on Pleistocene times (Costa et al., 1981). In coprolites and coprolite fragments, inclusions such as seeds and small pieces of tissues were identified, indicating the use of vegetables. The presence of these small fabric materials and seeds may indicate the archaeological material related to the Una burials culture.

Eggs of Capillariidae were found with diverse types of ornamentation, suggestive of the genera *Aoncotheca* or *Capillaria* (Table 2, Supplementary Table S2). Wild felines in Southeast Brazil, including *Leopardus gruttlus, L. pardalis, L. wiedii*, and *P. concolor* of Atlantic Forest, have been found infected with capillariid eggs (Nakamura, 2005; Quadros et al., 2009; Ventura-Morales et al., 2012). As apex predators, they hunt various animals across different habitats, potentially exposing themselves to parasitic infections. Following development in the soil, eggs become embryonated and thus infectious. Certain parasite species, such as *Calodium hepaticum*, infection arises when the host consumes fertilized eggs present in the environment, originating from contaminated feces (Quadros et al., 2016). Inside the host, the parasite matures and settles in the liver of animals and humans. Pseudoparasitism can occur when the host ingests carcasses of animals with infected livers. This involves consuming unembryonated eggs of prey, which pass through the digestive system of predators directly into the soil (Spratt, 2006; Carvalho-Costa et al., 2009).

According to IAB records, the coprolite positive for capillariid eggs determined to be from *D. albiventris* origin, was recovered during the salvage, the rescue of archaeological remains before the collapse of a close rock wall. Capillariidae have been reported in marsupials, including species of opossums, most commonly found in the Brazilian *Cerrado* biome from Rio Grande do Sul and Minas Gerais states. A second coprolite was characterized as from *P. onca* and exhibited the presence of a capillariid egg. With significant zoonotic potential, animals play a crucial role in the parasitic cycle of *C. hepaticum*. They are commonly associated with infections in canids, felids, swine, rodents, and primates (Reinhard et al., 2013; Côté et al., 2016; Bailly et al., 2019; Petrigh et al., 2021). Only one finding of capillariid eggs in coprolites from northeastern Brazil was reported. Sianto et al. (2014), identified various capillariid eggs in felid coprolite recovered from stratigraphic layers dating back up to 9,150 BP at the *Serra da Capivara* National Park. The diversity in the form and/or dimensions of eggs as a manifestation of phenotypic plasticity implies variation in the natural environment of capillariid hosts (Borba et al., 2021a). This phenomenon leads to the expression of diverse parasite phenotypes due to plasticity when a species infects different hosts (Poulin, 1996; Borba et al., 2021b). Following the study of Borba et al. (2021a), in the present study, we classified capillariids by the eggshell surface ornaments, geographical location, morphology, morphometry, and host. Eggs were isolated for morphological and morphometric analyses, identified through decision trees (DT) generated from databases of helminthological collections from the Collection de *Nématodes Zooparasites du Muséum National d’Histoire Naturelle de Paris* (MNHN/France) and *Coleção Helmintológica do Instituto Oswaldo Cruz* (CHIOC, IOC/FIOCRUZ/Brazil) with parameters of morphology including length and width, polar plug width and height, and shell thickness, host and geographical location of specimens, were included, along with AI/ML algorithms. The presence of capillariids eggs in jaguar and white-ear opossum coprolites can indicate false parasitism, due to the possibility of parasite acquisition through infected prey. The identification of capillariid parasites in these animal species is based on the understanding that the capillariid species detected are not typically known to directly infect jaguars or white-eared opossums. Instead, these parasites are more commonly associated with the prey species consumed by these predators. This suggests that the presence of capillariids in these hosts may be incidental, likely due to the ingestion of infected prey rather than a direct parasitic relationship. Furthermore, there is a significant gap in the current understanding of the life cycles of these capillariids within their various hosts, including the specific developmental stages and transmission dynamics of most capillariid species. This knowledge gap complicates efforts to fully assess the ecological and epidemiological roles these parasites play within the host community, highlighting the need for more detailed research on capillariid-host interactions in both definitive and intermediate hosts.

Employing the AI/ML decision trees considering all parameters (MM + H + GL) it was possible to identify the capillariid egg morphotype GGII01 as *C. venusta* if the prey was a bird, or *C. exigua* if the prey was a mammal. In the Brazilian *Cerrado* biome, there are more than 17 orders of birds, ranging from small Apodiformes, like the hummingbird, to large birds like the rhea (Costa et al., 1981). Free-living wild birds from Southeast Brazil were found parasitizing with *C. venusta* (Melo et al., 2021). Even though *C. exigua* typically parasitizes hedgehogs in Europe and not in the Americas, but its characteristics and measurements are still a match. The sample GGII15-01 is identified as *A. myoxinitelae*, a species that infects rodents. The Brazilian *Cerrado* biome has a vast diversity of rodents, ranging from large ones like capybaras to small rodents found throughout the extent of this Brazilian biome (Costa et al., 1981). GGII15-02 was identified as *A. pulchra* using all parameters of IA/ML. Cardia et al. (2014) identified the only record of *A. pulchra*, in eight bats of *N. laticaudatus* and *N. macrotis* in São Paulo state, southeast Brazil. In samples GGII33 and GGII51 *C. exigua*, was identified, however, there is no register of *C. exigua* in Brazil.

**Figure 4:**
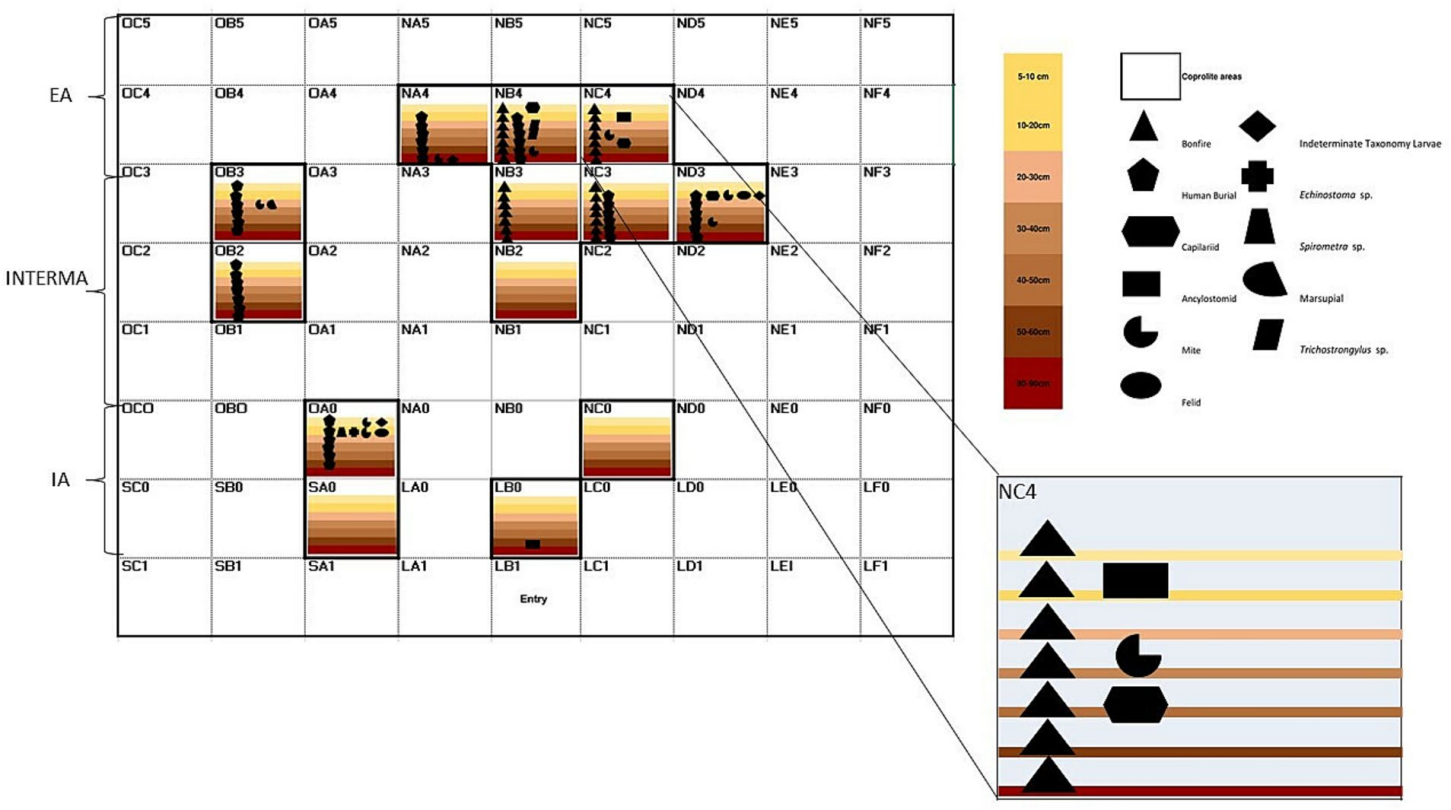
Integrative diagram of spatial and temporal analyses of the archaeological site GGII. Inside layers along with paleoparasitological and paleogenetic results. IA, internal area; INTERMA, intermediate; EA, external area. Layers are represented by different colors; Symbols are associated with paleoparasitological and paleogenetic results.

According to the integrative diagram of spatial and temporal analyses (Figure 4), the first stratigraphical layers contained the majority of coprolites studied in paleoparasitological and paleogenetic analyses. These layers were primarily located around the external north bonfire, dated up to 8,125 BP, and the main entryway. Parasites such as *Spirometra* sp. and *Echinostoma* sp. were identified in these areas. As the stratigraphic layers deepen (Figure 4), there are fewer coprolites identified. Human coprolites containing *Trichostrongylus* sp. eggs are present around the bonfire and entry area. Additionally, human burials were observed in these locations, indicating a significant level of activity and habitation. Despite this, parasitic remains are still found in the same areas, suggesting ongoing or recurring parasitic infections (Figure 4).

In the present study, humans were infected with *Trichostrongylus* sp. and ancylostomids, both geohelminth parasites that undergo a developmental phase in the soil, which differ in terms of egg morphology, morphometry, and host preference. Species of *Trichostrongylus* can parasitize diverse classes and species of animals, and Ancylostomids found in human remains indicate *N. americanus*, the most commonly observed in the New World (Hoagland and Schad, 1978). Ancylostomatidae parasites were found at sites spanning from the North to the South of Brazil, with the oldest dating back to 7,230 BP in the *Serra da Capivara* National Park, Piauí state, northeast Brazil (Araújo et al., 1988; Ferreira et al., 2011). In southeast Brazil, the presence of the parasite has been documented at the *Boqueirão Soberbo* site and, as mentioned, in the paleoparasitological investigations of sacral sediments and coprolites at the GGII site (Ferreira et al., 1989a; Iñiguez et al., 2022). Individuals infected with the larvae may suffer from symptoms including abdominal pain, nausea, and severe iron deficiency. Anemia arises from a heavy parasitic burden during persistent infection. The larvae feed on organic compounds, progress to the infective stage, and predominantly penetrate the epithelial tissue of barefoot individuals (Stoll, 1946). The hatching of the larvae directly correlates with soil temperature, type, and moisture percentage. In such conditions, the deposited egg in the soil undergoes development into the initial larval stage. The occurrence of ancylostomid eggs in human coprolites of the GGII site indicates that the population had a cultural practice of barefoot ambling since the infective larvae stage of this parasite is related to skin penetration, mainly through feet. Additionally, increasing population density, and the possibility that excrement was being deposited in communal spaces and areas with high human circulation (Hoagland and Schad, 1978; Yoshikawa et al., 2018).

The presence of indeterminate taxonomy larvae and mites in some of the samples requires further investigation, as the expertise of a taxonomic specialist is essential for the identification of these specimens. Subsequent molecular analyses will be necessary to determine whether these organisms are free-living or represent a parasitic stage in their evolutionary cycle. Ferreira et al. (1980, 1983) reported the presence of helminth larvae from GGII samples identified. These larvae could potentially be attributed to helminths, as similar findings have been documented in prior studies. There is still a possibility that the indeterminate taxonomy larvae identified in some of the GGII coprolites may have contributed to further degradation of the coprolite itself and the organic material, including the parasite eggs themselves, as observed in the work of Camacho et al. (2018). Camacho et al. (2018) studied the association between the occurrence of mites, nematode larvae, and the degradation of parasite eggs in coprolites from North American archaeological sites. In the study, the authors discuss that the main taphonomic element affecting egg preservation is ecological factors, and how the presence of mites and fungi suggests a potential impact on the morphology of the eggs.

By integrating studies of contemporary animal behavior with archaeological content, it becomes possible to reconstruct movements, ecosystems, biological, and ecological relationships (Shillito et al., 2020). In paleoecological studies, unlike modern ecological analyses, the study environment is determined before examining the biotic components. Consequently, in paleoenvironmental investigations, there is a greater emphasis on understanding the entire abiotic component rather than the specific individuals from an archaeological site (Bottjer, 2016). During the analysis of the GGII site, it was no different. We knew about the environment of the archaeological site long before understanding which individuals would be studied. Archaeological research in the Pleistocene has revealed that the communities inhabiting the central region of the *Cerrado* biome experienced environmental changes, leading to the search for different types of sustenance/food to ensure survival (Ledru et al., 2006). Despite the flora environment changing during the transition from the Pleistocene to the Holocene in the *Cerrado* biome, the fauna has remained the same until nowadays. The *Cerrado* fauna contains a great richness of animals, including birds from over 20 different classes, mammals ranging from Rodentia to Perissodactyla, and reptiles from different habitats (Costa et al., 1981). In this study, we were able to gain a better understanding of the interaction of these animals with the local human population through the identification of parasites. The horticulturists of GGII possessed complex baskets and adornments made of aquatic bivalves, indicating that this population possibly consumed local Mollusca, as well as the presence of nearby bodies of water demonstrating possible fish consumption. The bones of small mammals near the fireplaces also provide insight that they could have fed on small mammals identified in the southeastern Brazilian *Cerrado* Bioma, such as marsupials of the genus *Didelphis* and rodents of the genus *Rattus*, animals that can also be hosts to trichostrongylids, capillariids, and species of *Echinostoma*, and *Spirometra* (Bottjer, 2016; Wilson and Mandrak, 2021).

**Figure 5:**
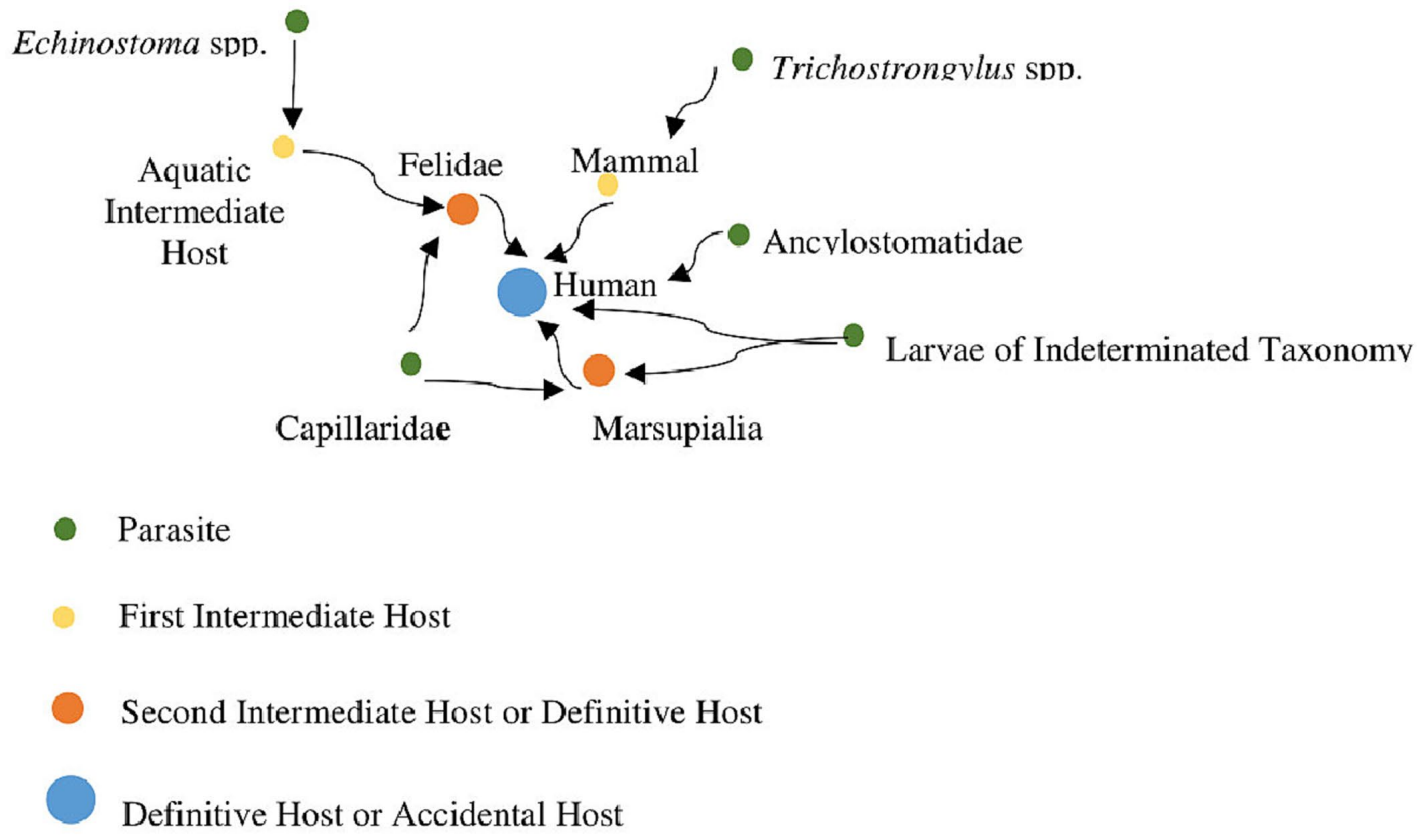
Network data of hosts and parasites recovered from coprolites of GGII. Hosts = 8 Humans, 2 Felids, 2 Marsupials. Parasites = Capillariidae (Felid and Marsupial), Ancylostomatidae (Human), *Trichostrongylus* sp. (Human), *Spirometra* sp., *Echinostoma* sp. (Felid), and Larvae of indeterminate taxonomy (Human and Marsupial).

In GGII, the first epidemiological transition according to Omran (1971) can be observed. During the Pleistocene, the means of subsistence for the human population was that of hunter-gatherers, as indicated by archaeological records. The Holocene is marked by the appearance of evidence of the Una tradition, with traces of horticultural subsistence. The study of epidemiological transitions provides us insights into cultural changes, diets, and subsistence, as well as the composition of parasites in a population. In the case of GGII, we can observe that parasites like trichostrongylids were identified in the older horizon, associated with hunter-gatherers, while in the horticultural horizon, parasites like hookworms were observed. Species of *Trichostrongylus* are zoonotic parasites commonly found in ruminants and wild animals (Santiago et al., 1975; Radavelli et al., 2014), which are more associated with a free-living lifestyle, similar to that of hunter-gatherers. This suggests that these ancient human populations may have had more contact with wild animals during earlier periods. On the other hand, in the horticultural horizon, the human population began to transition away from a nomadic lifestyle, as evidenced by the presence of domesticated plants and the construction of ceramics of the Una tradition. In this horizon, human-specific parasites like hookworms were found, indicating a possible increase in the human population within the cave and a move towards a more sedentary lifestyle. These data may suggest an epidemiological transition in the populations that frequented this archaeological site during both subsistence horizons (Figure 5).

Archaeologists at the GGII site have reported the presence of animal bones near bonfires, related to rodents and small mammals, which are distributed across the internal, intermediate, and external areas of the site (Sene, 2007). Given the ceremonial nature of the GGII site, these findings may be associated with the cultural and religious practices, either during ceremonial rituals or during the individual feeding activities of the human population that utilized the site. Additionally, the bones of small animals identified during the archaeological analysis may have originated from animals hunted for human consumption or be the prey of larger animals (Sene, 2007; Seda et al., 2011). This information is consistent with the identification of animal remains through paleogenetic analysis of GGII, demonstrated in this study. These animals could have entered the cave in search of carcasses or food remnants. Moreover, they could have been the very animals hunted by humans and used in rituals or for sustenance. Further analyses are necessary with a larger sample size and a more sensitive approach, such as palaeogenomics. GGII coprolites have shown considerable potential for unveiling insights into the paleoecological history of the seasonal inhabitants and achieving a richer understanding of the animal and human dynamics, diet, and daily life of the GGII people.

## 5 Conclusion

*Gruta do Gentio* II site hosts a vast diversity of coprolites, indicating that not only humans but distinct species of animals were infected with zoonotic parasites that circulated in the cave between 3,490 and 8,125 BP, the dating of the stratigraphical layers where coprolites collected were positive. We suggested that an ecological progression and a non-overlapping site recurrence between humans and mammal species may have happened in both subsistence horizons, the hunter-gatherer, and the horticulturists. Not only human coprolites, but various mammal coprolites were identified throughout the entire cave, within human burials, cultural materials, bonfires, and animal bones. The identification of *Panthera onca, Didelphis albiventris, Puma concolor,* and *Philander opossum* coprolites indicates the presence of these animals in the GGII site in agreement with the morphology and morphometry of coprolites and the wildlife identified in the region. The preservation of coprolites, and parasites, as well as the efficiency of the molecular approach for host identification, are relevant parameters for the understanding of the paleoecological scenery of the GGII site.

The paleoparasitological examination exhibited a diversity of enzootic and zoonotic parasitic helminths, exemplified by species of Capillariidae, *Trichostrongylus* sp., Ancylostomatidae, *Spirometra* sp., and *Echinostoma* sp. Additionally, the paleoparasitological analysis of coprolites elucidated the temporal and spatial circulation of various animals, including humans. The application of multidisciplinary studies is increasingly employed to enhance and strengthen scientific research, providing meaningful data for scientific discourse. The present study integrates paleoparasitology, paleogenetic, and archaeological data of the GGII site to understand not only the parasite infections but also the way of life, culture, and diet of animals and humans. In the current study, it was observed that in pre-colonial Southeast Brazil, the parasites were not necessarily different during the first epidemiological transition. However, through the presence of identified parasitic species, we were able to observe health, way of life, and interactions among animal and human populations frequenting GGII.

## Data Availability

The datasets presented in this study can be found in online repositories. The names of the repository/repositories and accession number(s) can be found in the article/Supplementary material.

## Footnotes

^1^ http://blast.ncbi.nlm.nih.gov/Blast.cgi

^2^ https://earth.google.com
