# Supplementary material for: Integrating paleoparasitological, paleogenetic, and archaeological data to understand the paleoecological scenario of pre-Columbian archaeological site Gruta do Gentio II, Brazil
Source: Front Microbiol. 2025 Jan 20;15:1505059. doi: 10.3389/fmicb.2024.1505059 (PMC11788412; doi:10.3389/fmicb.2024.1505059)
Supplement: Supplementary file 1 [file Data_Sheet_1.docx]

Supplementary Material

**Integrating Paleoparasitological, Paleogenetic, and Archaeological Data to Understand the Paleoecological Scenario of pre-Columbian Archaeological Site Gruta do Gentio II, Brazil**

**GURJÃO, L, L ¹; BRITO L, S ¹; DIAS, O ^2^; NETO, J^2^; IÑIGUEZ A. M.*¹^,^**

*** Correspondence:** Corresponding Author: [alena@ioc.fiocruz.br](mailto:alena@ioc.fiocruz.br)

## Supplementary Tables

| CoproID | Total Mass | Weight Rehydration |
| --- | --- | --- |
| GG01 | 21.428g | 5.063g |
| GG02 | 20.449g | 5.293g |
| GG04 | 83.665g | 5.729g |
| GG05 | 28.786g | 5.208g |
| GG06 | 10.950g | 5.470g |
| GG13 | 5.631g | 5.631g |
| GG15 | 2.962g | 2.962g |
| GG30 | 1.553g | 1.553g |
| GG31 | 0.815g | 0.815g |
| GG32 | 6.871g | 5g |
| GG38 | 1.032g | 1.032g |
| GG41 | 0.349g | 0.349g |
| GG46 | 0.332g | 0.332g |
| GG47 | 4.976g | 4.976g |
| GG48 | 0.856g | 0.856g |
| GG52 | 0.965g | 0.965g |
| GG58 | 1.163g | 1.163g |
| GG63 | 3.471g | 3.471g |
| GG74 | 19.350g | 5.057g |
|  |  |  |

**Supplementary Table S1.** Total and utilized mass of the selected coprolites following morphology and morphometry of mammals according to Chame (2003). Succeeding by the weight rehydration, correlated to the total weight of the coprolite or coprolite segments used to submit to rehydration procedures of paleoparasitology.

| Parasite | Eggs per Taxa | Eggs per Coprolite | Eggs per Slide | Mean Length | Mean Width | SD  Length | SD  Widht |
| --- | --- | --- | --- | --- | --- | --- | --- |
| Ancylostomatidae | 1 | 1 per 1 | 1 per 1 | 67.453 | 34.913 | - | - |
| *Trichostrongylos* sp. | 2 | 2 per 1 | 2 per 1 | 82.2995 | 45.01 | 3.09 | 2.4 |
| *Echinostom*a sp. | 2 | 2 per1 | 1 per 1 | 58.39 | 111.159 | 17.2 | 7.12 |
| *Spirometra* sp. | 5 | 5 per 1 | 1 per 1 | 38.89 | 62.2722 | 2.167 | 4.31 |
| *Capillaria venusta* | 1 | 1 per 1 | 1 per 1 | 51.672 | 25.032 | - | - |
| *Aoncotheca myoxinitelae* | 3 | 3 per 1 | 3 per 1 | 68.073 | 57.280 | 7.05 | 5.6 |
| *Aoncotheca pulchra* | 3 | 3 per 1 | 3 per 1 | 53.264 | 44.490 | 8.5 | 5.7 |

**Supplementary Table S2**. Paleoparasitological results of coprolites identified in stratigraphical layers of GGII. Information about eggs per taxa, coprolites, slide, mean length and width, and SD. Measures in µm. The mean refers to the sum of all the measurements of length and width of the identified eggs, divided by the number of identified specimens. The standard deviation (SD) refers to the degree of variation or dispersion within the dataset of length and width of the identified eggs.

| Parasite Infection | Capillariidae | Ancylostomatidae | *Trichostrongylus* sp. | *Echinostoma* sp*.* | *Spirometra* sp. |
| --- | --- | --- | --- | --- | --- |
| Transmission | Ingestion of viscera or soil contaminated.  with infective egg | Penetration through Tissue | Ingestion of water or food containing  the infective larvae stage | Ingestion of second intermediate host  containing infective cyst | Ingestion of second intermediate host  containing infective larvae |
| First Intermediate Host | NA | NA | NA | Aquatic Invertebrate | Aquatic Invertebrate |
| Second intermediate Host | NA | NA | NA | Mammal, bird, or reptile | Mammal, bird, or reptile |
| Definitive Host | Wild Host Range | Mammals | Mammals | Mammals | Mammals |

**Supplementary Table S3.** Transmission, first intermediate host, second intermediate host and definitive host of each parasite egg identified during de paleoparasitological analysis of GGll. NA – Not applicable. The transmission dynamic table, including transmission and hosts by parasite, was constructed inspired by the infographics located in Slifko et al., 2000.
